# Defining the fitness of HIV-1 isolates with dual/mixed co-receptor usage

**DOI:** 10.1186/s12981-015-0066-7

**Published:** 2015-10-03

**Authors:** Immaculate L Nankya, Denis M Tebit, Awet Abraha, Fred Kyeyune, Richard Gibson, Oyebisi Jegede, Gabrielle Nickel, Eric J Arts

**Affiliations:** Division of Infectious Diseases, Department of Medicine, Case Western Reserve University, 10900 Euclid Ave., Cleveland, OH 44106 USA; Virology Program, Department of Molecular Biology and Microbiology, Case Western Reserve University, Cleveland, OH USA; Joint Clinical Research Centre, Kampala, Uganda; Myles Thaler Center for AIDS and Human Retrovirus Research, Department of Microbiology, Immunology and Cancer Biology, University of Virginia, Charlottesville, VA USA; Laboratory of Virology and Infectious Disease, Rockefeller University, New York, USA

**Keywords:** HIV-1 fitness, Co-receptor usage, Entry inhibitors

## Abstract

**Background:**

CCR5-using (r5) HIV-1 predominates during asymptomatic disease followed by occasional emergence of CXCR4-using (x4) or dual tropic (r5x4) virus. We examined the contribution of the x4 and r5 components to replicative fitness of HIV-1 isolates.

**Methods:**

Dual tropic r5x4 viruses were predicted from average HIV-1 env sequences of two primary subtype C HIV-1 isolates (C19 and C27) and from two patient plasma samples (B12 and B19). Chimeric Env viruses with an NL4-3 backbone were constructed from the B12 and B19 env sequences. To determine replicative fitness, these primary and chimeric dual tropic HIV-1 were then competed against HIV-1 reference isolates in U87.CD4 cells expressing CXCR4 or CCR5 or in PBMCs ± entry inhibitors. Contribution of the x4 and r5 clones within the quasispecies of these chimeric or primary HIV-1 isolates were then compared to the frequency of x4, r5, and dual tropic clones within the quasispecies as predicted by phenotypic assays, clonal sequencing, and 454 deep sequencing.

**Results:**

In the primary HIV-1 isolates (C19 and C27), subtype C dual tropic clones dominated over x4 clones while pure r5 clones were absent. In two subtype B chimeric viruses (B12 and B19), r5 clones were >100-fold more abundant than x4 or r5/x4 clones. The dual tropic C19 and C27 HIV-1 isolates outcompeted r5 primary HIV-1 isolates, B2 and C3 in PBMCs. When AMD3100 was added or when only U87.CD4.CCR5 cells were used, the B2 and C3 reference viruses now out-competed the r5 component of the dual tropic C19 and C27. In contrast, the same replicative fitness was observed with dualtropic B12 and B19 HIV-1 isolates relative to x4 HIV-1 A8 and E6 or the r5 B2 and C3 viruses, even when the r5 or x4 component was inhibited by maraviroc (or AMD3100) or in U87.CD4.CXCR4 (or CCR5) cells.

**Conclusions:**

In the dual tropic HIV-1 isolates, the x4 replicative fitness is higher than r5 clones but the x4 or x4/r5 clones are typically at low frequency in the intrapatient virus population. Ex vivo HIV propagation promotes outgrowth of the x4 clones and provides an over-estimate of x4 dominance in replicative fitness within dual tropic viruses.

**Electronic supplementary material:**

The online version of this article (doi:10.1186/s12981-015-0066-7) contains supplementary material, which is available to authorized users.

## Background

Human immunodeficiency virus entry into host cells involves binding to CD4 and one of two coreceptors, namely CCR5 and CXCR4, hence the two viral phenotypes, the r5- and x4-using viruses. CCR5 is expressed in conjunction with surface CD4 on activated lymphocytes, macrophages, dendritic cells, and brain cells, while CXCR4 with CD4 are found on the surface of resting T cells and monocytes [1–3]. Although other chemokine receptors can mediate low level HIV-1 entry [4, 5], CCR5 and CXCR4 remain the primary co-receptors for all diverse HIV-1 subtypes as well as other primary lentiviruses, e.g. HIV-2, simian immunodeficiency virus from chimpanzees, sooty mangabeys, and African green monkeys [6–9]. The r5 HIV-1 variants are preferentially transmitted and predominate during the asymptomatic period of infection whereas x4 HIV-1 emerges late in disease and in less than 50 % of patients [10–13].

HIV-1 entry into host cells is almost exclusively triggered by Env glycoproteins. In fact, expression of only HIV-1 Env in an effector cell can mediate fusion with a target cell expressing CD4 and a co-receptor (reviewed in [14]). During virus assembly, the Env gp160 precursor is cleaved by cellular proteases into the membrane spanning gp41 and the soluble gp120 subunits, which remain covalently linked through a disulfide bridge [15]. On the virus particle, Env gp120/gp41 form trimer spikes that mediate virus attachment to the receptor complex on the cell surface (reviewed in [14]). First, a conserved C4 region in gp120 interacts with the amino-terminal extracellular immunoglobulin-like domain of the CD4 receptor. Upon interaction with CD4, the V3 loop of gp120 can engage the second extracellular loop of CCR5 (or CXCR4) while a gp120 conformational change exposes a second Env site (comprised in part by the C2 region) to bind with the N terminus of the co-receptor.

Although the V3 loop appears to define selective binding of either CCR5 or CXCR4, specificity for a particular co-receptor also appears to extend to other regions throughout gp120 [16–18]. Specifically, the V1/V2 loops on HIV-1 gp120 can influence co-receptor usage but not as efficiently as the V3 domain [19–21]. Differential interaction with the co-receptors may be related to the higher negative charge of the second extracellular loop of CXCR4 compared to that of CCR5, which may in turn favor binding to a more positively charged gp120 V3 loop [22, 23]. Several algorithms/models have been developed to predict HIV-1 co-receptor usage from the amino sequence in Env. Geno2Pheno and PSSM are algorithms based solely on the V3 loop sequence and are generally greater than 85 % accurate in predicting co-receptor usage by HIV-1 subtype B strains [24–27]. Both of these models are heavily weighted by positive charges at amino acid positions 11 and/or 25 in the V3 loop, which are nearly absolute in predicting CXCR4 usage. In addition, Phenoseq a new program has the ability to predict coreceptor usage for all the major subtypes.

The switch from r5 to x4 virus has been associated with rapid depletion of CD4+ T cells [28, 29]. Patients harboring x4 viruses progress to AIDS more rapidly than those harboring exclusively r5 viruses implying that CXCR4 utilization is linked to a stronger pathogenic phenotype and a switch to CXCR4 utilization is a causative factor in disease progression, this has been mainly studied in subtype B [28, 30, 31]. However, this hypothesis is quite controversial considering that CD4 cell decline is observed in nearly all HIV-infected patients despite the fact that only 50 % switch from r5 to x4 viruses and only late in disease among subtype B individuals. A switch in coreceptor usage rarely occurs in subtype C. Three main hypotheses for the switch in coreceptor usage have been proposed. The transmission-mutation hypothesis proposes that predominance of r5 viruses during transmission and early infection is due to active selection of r5 and that the coreceptor switch is a result of virus evolution during the course of disease [29]. The target cell-based hypothesis is based on the inability of quiescent, naïve CXCR4+/CD4+ T cells to support HIV-1 replication whereas the memory CCR5+/CD4+ T cells are activated at a higher frequency and as such, support greater HIV-1 replication [32, 33]. With the depletion of the memory T cell population, there would be a shift in cells that support x4 versus r5 replication. Finally, the immune-control hypothesis assumes that x4 viruses are recognized better by the immune system than r5 viruses [34, 35]. Thus, x4 viruses would only emerge later in infection due in part to poor virologic control by a dysfunctional immune response. This hypothesis also suggests that emergence of x4 over r5 variants relates to higher replicative fitness [36]. However, increased sensitivity of CXCR4-using virus compared to CCR5-using virus to autologous neutralizing antibodies is not supported by other studies [37].

These hypotheses generally focus on the displacement of r5 HIV-1 by x4 HIV-1 late in disease progression but do not necessarily account for dual tropic HIV-1 variants. An HIV-1 isolate that utilizes both CCR5 and CXCR4 for entry is commonly referred to as dual tropic (r5x4). However, the intrapatient dual/mixed (dm) may be any combination of HIV-1 clones using just CCR5 or CXCR4 for entry (x4 or r5 HIV-1) in addition to clones capable of infecting both CD4+/CCR5+ and CD4+/CXCR4+ cells (r5x4 HIV-1). The contribution of the r5, x4, and r5x4 to the replicative fitness of an HIV-1 isolate is poorly understood but could be consequential to disease progression. Dominance of pure x4 variants within the intrapatient HIV-1 population at any time, even in late disease is relatively rare. Instead, most HIV-1 isolates that appear to prefer CXCR4 for host cell entry still retain the ability to infect CD4+/CCR5+ cells.

In previous studies, we performed hundreds of competitions between HIV-1 isolates of all group M subtypes with a focus of subtype C strains [38–40]. Subtype C isolates were substantially less fit than all other group M isolates when competed in head-to-head pair-wise competitions in PBMC cultures using a set of r5 and x4 HIV-1 isolates [40]. Two subtype C HIV-1 isolates (C19 and C27) capable of infecting CD4+/CXCR4+ cells were dual tropic (i.e. also capable of infecting CD4+/CCR5+ cells). Thus, the reduced fitness of at least two of these “x4” subtype C isolates may be related to the r5x4 phenotype, especially if there was a high proportion of pure r5 HIV-1 isolates in the C19 and C27 HIV-1 isolates. In almost all cases, x4 HIV-1 have faster replication kinetics than r5 HIV-1 isolates when performing dual infections of PBMCs [40, 41]. This dual infection is not a “true” competition considering that the r5 and x4 viruses likely replicate in different susceptible cells within the PBMC population. In this study, we examined the impact of both the “CXCR4”-using and “CCR5”-using clones to the replicative fitness of r5x4 HIV-1 isolates in CD4+/CCR5+ cells, CD4+/CXCR4+ cells, and in the mixed cell population found in PBMCs. We also competed the r5x4 HIV-1 isolates against r5 or x4 primary HIV-1 isolates in the presence of AMD3100 (CXCR4 antagonist) or MVC (CCR5 antagonist) to further confirm relative fitness of each phenotype in the dual tropic HIV-1 isolates. Finally, the contribution of the r5 and x4 component to replicative fitness of the primary HIV-1 isolates was compared to the sum of its parts by characterizing or predicting the co-receptor usage of the representative clones in these dual tropic virus populations.

Dual tropic and dm viruses are commonly observed in HIV infected patients and are frequently responsible for failure of maraviroc treatment. Treatment failure of maraviroc can result from the emergence of resistant CCR5 (r5)- using viruses with reduced susceptibility to MVC [42–45]. The contribution of the CCR5(r5)- and CXCR4(x4)-using component of the intrapatient HIV-1 population to overall virus replication in susceptible cells is poorly understood. Each component of the dual tropic and dm virus can replicate in different T cell and macrophage subsets. In this study, we have determined the contribution of the r5-using and x4-using components of dm and dual tropic HIV on replicative fitness and determine if a specific phenotype may be dominant in disease.

## Results

### Identification of dual tropic HIV-1 isolates from patient samples

**Fig. 1:**
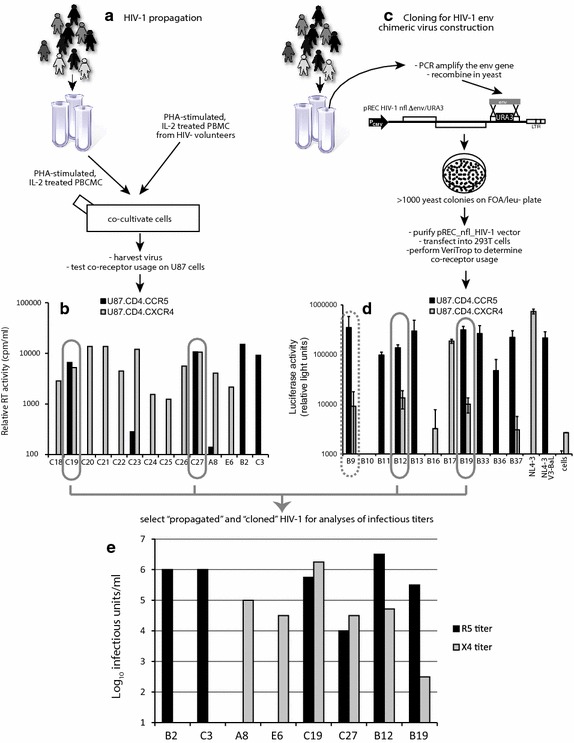
Identification of dual/mixed isolates following HIV-1 propagation or construction of env chimeric viruses. **a** HIV-1 subtype C isolates from Zimbabwe were propagated by PBMC co-cultivation as described [46] and in the “Methods”. **c** The HIV-1 env coding region of HIV-1 subtype B infected patients was PCR amplified and then cloned into pREC_nfl_HIV-1_nl4-3_Δgp120/URA3 via yeast recombination/gap repair [47]. The propagated HIV-1 isolates (**a)** and cloned env genes expressed following 293T transfection (**c)** were screened for co-receptor usage by infection(1) (**b**) or cell-to-cell fusion (**d**) (Veritrop; [48]) on U87.CD4 cells expressing either CXCR4 or CCR5(2). In the virus infection system (**b**), virus production was monitored by RT activity in supernatant [87]. In the cell-to-cell fusion assay (**d**), the ability of pREC_nfl_env_ptX_ to modulate receptor binding and cell fusion was monitored by firefly luciferase activity, i.e. Rev/Tat in the effector cell controlling Luc expression under the control of the HIV-1 LTR and rescued via the RRE housed in an intron with Luc [80]. Two primary HIV-1 isolates and two env chimeric viruses were propagated to measure infectious titers on PBMCs (Additional file 1: Table S1), U87.CD4.CCR5 and U87.CD4.CXCR4 cells (**e**) using the classical Reed–Munch approach [79].

Two methods were employed to isolate HIV-1 from patient samples and then screen for viral tropism. The first method involved initial co-cultivation of PBMCs from an HIV-infected patient with PBMCs from an HIV-negative donor (Fig. 1a). These standard co-cultivations were performed on a set of patient samples from Zimbabwe as previously described [40]. This initial analysis screened over 28 HIV-1 isolates and found 10 that were syncytium inducing (SI) and capable of replicating on MT2 cells [46]. These primary SI HIV-1 were then equalized for RT activity (see “Methods”) and added to U87.CD4 cells expressing either CCR5 or CXCR4 [40] (Fig. 1b). Two (C19 and C27) of 10 primary HIV-1 isolates were dual tropic, i.e. capable of infecting and replicating in U87.CD4 cells expressing either CXCR4 or CCR5 as a co-receptor. HIV-1 C23 showed CCR5 (r5) just above the limit of detection whereas the remaining strains in this cohort were CXCR4 (x4) as previously described.

As part of another study that screened for co-receptor usage, HIV-1 env chimeric viruses were produced by homologous recombination in yeast rather than propagating HIV-1 from patient samples (Fig. 1c). The yeast-based recombination/gap repair cloning method can efficiently replace the yeast URA3 gene with the gp120 coding region of the env gene in our pREC_nfl_HIV-1_NL4-3_Δgp120/URA3 vector (see “Methods”) [44, 47]. The efficiency of this cloning method limits the genetic bottleneck introduced by prolonged PBMC co-cultivation and subsequent virus propagations. Following env cloning, the pREC_nfl_env_Ptx_ vectors from each patient sample (Ptx—patient x) were purified from pooled yeast colonies (>1,000) and transfected into 293T cells. We have recently described an efficient cell-to-cell fusion assay to screen for co-receptor usage using the U87.CD4.CXCR4 or U87.CD4.CCR5 as target cells [48]. In our preliminary screen of 11 patient-derived env chimeric viruses, we identified three dual tropic HIV Envs (B9, B12, and B19), five r5s, and two x4s (Fig. 1d). The pREC_nfl_env vectors of B9, B12, and B19 were co-transfected with the complementing vector (pCMV_cplt) to produce replication-competent chimeric HIV as described [47]. This virus was then used to infect U87.CD4.CXCR4 and U87.CD4.CCR5 cells to confirm co-receptor usage. HIV-1 env_B9 did not replicate in either cell line.

The x4 and r5 titers of each dual tropic primary and dual tropic env chimeric HIV-1 isolate along with a set of reference HIV-1 isolates were measured by standard TCID_50_ assays on U87.CD4.CXCR4 and U87.CD4.CCR5 cells, respectively (Fig. 1e). The dual tropic C19 and C27 viruses had equal infectious r5 and x4 titers whereas the dual tropic env_B12 and env_B19 viruses had higher r5 than x4 titers. Interestingly, the infectious titers derived from TCID_50_ assays using HIV-negative PBMCs was the same as the highest infectious titers from either U87.CD4.CXCR4 or U87.CD4.CCR5 cells. For at least the C19 and C27 viruses, the IU/ml on PBMCs did not reflect the simple addition of r5 and x4 infectious units suggesting that the clones within these dual tropic HIV-1 isolates may be primarily dual tropic rather than a mix of r5- and x4-only HIV-1 clones (Additional file 1: Table S1).

### Phylogenetic analyses of the dm HIV-1 isolates and env chimeric viruses

**Fig. 2:**
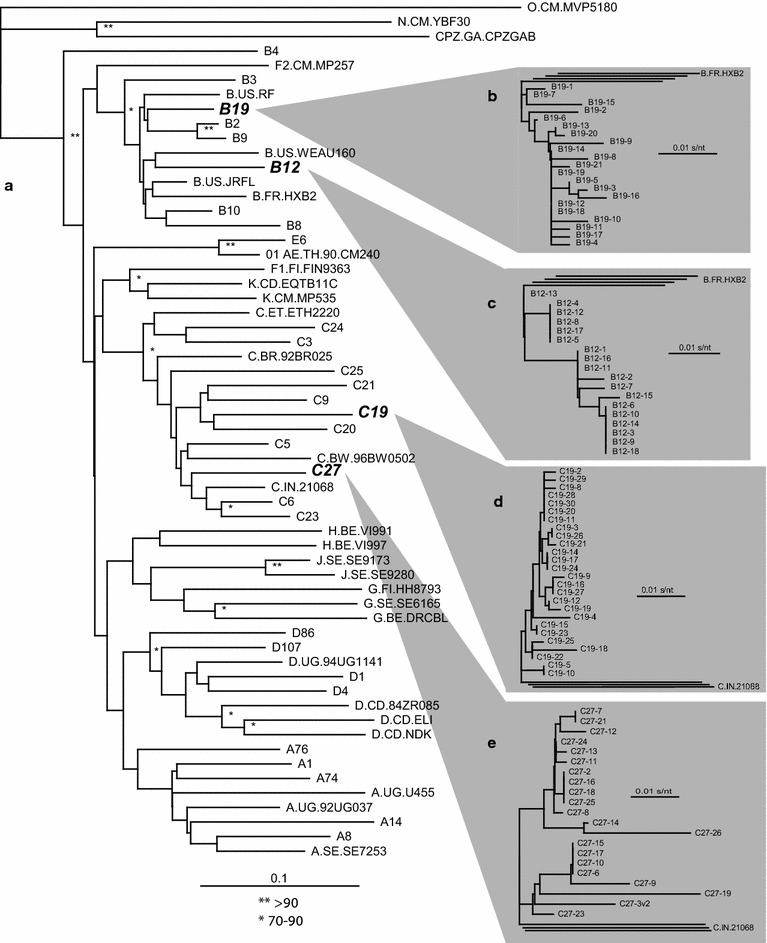
Neighbor joining phylogenic trees of two dual tropic HIV-1 isolates, two HIV-1 env chimeric viruses, and of the sampled clones in their virus population. Alignments and phylogenetic trees were constructed for the C2-V3 region (350 nt) of the four reference strains, r5 B2, r5 C3, x4 A8, and x4 E6, the two dual tropic HIV-1 isolates (C19 and C27), the two HIV-1 env chimeric viruses (B12 and B19) and set of reference strains (**a**). As described in the “Methods”, the Env coding region of two dual/mixed (dm) HIV-1 isolates were PCR amplified and cloned into pREC env via yeast-based recombination. The C2-V3 sequences from 21 B19 (**b**), 18 B19 (**c**), 26 C19 (**d**), 21 © clones (**e**) were aligned using MUSCLE and then schematic represented in phylogenetic trees.

Alignment of the env sequences confirmed that the bulk populations of the dual tropic C19 and C27 were subtype C whereas dual tropic env_B12 and env_B19 aligned with other subtype B HIV-1 sequences (Fig. 2a). In addition, the B12, B19, C19, and C27 viruses were not clonal but rather a population of related env sequences, based on approximately 10–15 sequenced clones from each (Fig. 2b–e).

### Contribution of x4 and r5 variants to replicative fitness in dual tropic and mixed r5x4 primary HIV-1 isolate

In a previous study, we performed two pairwise competition experiments in PBMC cultures with 14 primary r5 and 15 x4 HIV-1 isolates [40]. The phylogenetic relationships of these are shown in Fig. 2. To summarize, both the r5 and x4 HIV-1 subtype C isolates were less fit than the r5 and x4 HIV-1 isolates (respectively) of different group M subtypes. The poor replicative fitness of subtype C isolates has now been observed in over 2,000 head-to-head competitions with over 40 primary subtype C HIV-1 isolates [38–40, 49]. Although ten x4 HIV-1 subtype C isolates were dramatically less fit than any other x4 HIV-1 isolate, we also noted that two isolates, C19 and C27 were of a dual tropic phenotype and capable of infecting both CD4+/CCR5+ and CD4+/CXCR4+ susceptible cells [40]. When competing r5 against x4 HIV-1 isolates in PBMCs, the x4 viruses, regardless of subtype, had higher replicative fitness than an r5 HIV-1 isolate [40]. Thus, it is possible that reduced fitness of dual tropic C19 and C27 HIV-1 isolates may have been related to high proportion of r5 HIV-1 clones within these viruses.

To determine the relative contribution of the CCR5-using and CXCR4-using phenotypes in these dual tropic r5x4 HIV-1 isolates, the r5x4 C19 and C27 HIV-1 isolates were competed against four reference HIV-1 isolates, the r5-using B2 and C3 primary isolates and the x4-using A8 and E6 viruses (Fig. 1e). The control x4 A8 virus is characterized as having a high replicative fitness whereas the x4 E6 is of lower replicative fitness when compared in direct competition with other x4 isolates [39, 40]. Our control r5 B2 virus was also selected based on a higher replicative fitness as compared to other r5 HIV-1 isolates whereas r5 C3 has low replicative fitness [39, 40]. First, the titers of dual tropic C19 and C27 viruses as well as control x4 A8 and E6 HIV-1 were calculated using both PBMCs (“full” titer) and U87.CD4.CXCR4 (“x4” titer) for TCID_50_ assays (Additional file 1: Table S1).

**Fig. 3:**
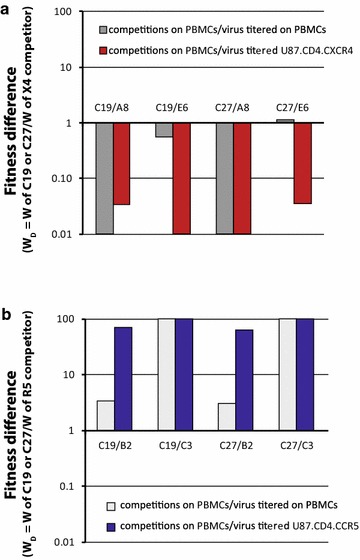
Relative replicative fitness of the dual tropic C19 and C27 HIV-1 when competed against reference strains on PBMCs. **a** The dual tropic HIV-1 isolates, C19 and C27 were competed against the x4 reference strains, A8 and E6 on PBMC using equal MOIs (0.004) of each virus tittered on U87.CD4.CXCR4 cells or PBMCs (same donor and blood draw as those used in competition). **b** A similar set of competitions on PBMCs involved the same dm viruses competed against the r5 reference strains, B2 and C3 using equal MOIs tittered on U87.CD4.CCR5 cells or PBMCs. Fitness difference (or ratio) is shown with all competitions where the relative fitness of the dual tropic HIV-1 isolate is plotted as a ratio to the relative fitness of the reference virus in the competition. The titers of all dual tropic and reference HIV-1 isolates are shown in Additional file 1: Table S1.

Direct competitions showed that the dual tropic C19 and C27 HIV-1 were completely outcompeted by A8 but still could compete with the x4 E6 HIV-1 (Fig. 3a), when virus inoculums were equalized based on PBMC titers. When inoculums were equalized based on x4 titers, the fitness of dual tropic C19 and C27 further decreased in competition with x4 E6 (Fig. 3a). The dual tropic C19 and C27 were then competed in PBMCs against the r5 B2 and C3 control viruses using the equalized “full” titers determined on PBMCs or the equalized “r5” titers determined on U87.CD4.CCR5. As observed with nearly all x4-using viruses, the dual tropic C19 and C27 HIV-1 isolates were more fit than the r5 C3 virus when inoculating with the PBMC titers (Fig. 3b). However, dual tropic C19 and C27 were only slightly more fit than the r5 B2 HIV-1. The “CXCR4”-using component of the dual tropic HIV-1 isolates are not necessarily in competition with these r5 HIV-1 in PBMC since they likely infect different susceptible cells in the PBMC population. Competitions with the control r5 B2 HIV-1 suggest a low frequency of an r5 phenotype in the dual tropic C19 and C27 viruses. When using U87.CD4.CCR5-derived titers, it was not surprising that C19 and C27 could further out replicate the r5 B2 and C3 viruses (Fig. 3b) in competition considering the fivefold increase in C19 and C27 inocula to equalize their “r5” titer with the titer of pure r5 B2 and C3 viruses.

**Fig. 4:**
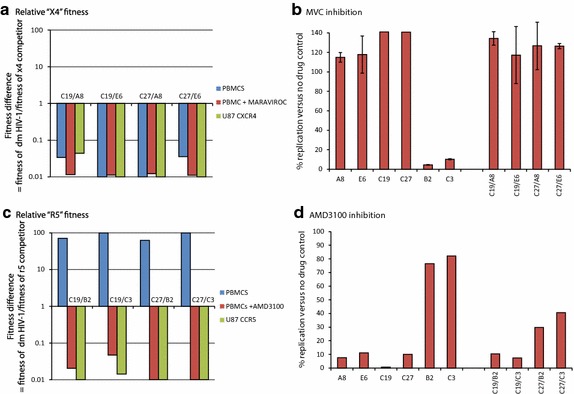
Fitness of the dual tropic HIV-1 isolate when blocking the virus component using CCR5 or CXCR4. **a** The primary dual tropic HIV-1 isolates were competed against the x4 [50] titereds (all titered on U87.CD4.CXCR4 cells) in U87.CD4.CXCR4 cultures or in PHA-stimulated, IL-2 treated PBMC cultures with or without maraviroc. The fitness differences are plotted as described in Fig. 2 and in the “Methods”. **b** The level of maraviroc inhibition of the monoinfections or dual infections was measured by relative RT activity in the cell free supernatant and plotted as a percentage of the no drug control. **c** The primary dual tropic HIV-1 isolates and r5 reference strains were also titered on U87.CD4.CCR5 cells and then competed together in PHA-stimulated, IL-2 treated PBMC cultures with or without AMD3100. The same dual infection with the same MOI (0.004) was repeated in U87.CD4.CCR5 cultures. The fitness differences are plotted as described in Fig. 2 and “Methods”. **d** The level of AMD3100 inhibition of the monoinfections or dual infections was measured by relative RT activity in the cell free supernatant and plotted as a percentage of the no drug control. All experiments were performed in triplicate with the exception of **c**, **d** (performed in duplicate) due to limited supply of AMD3100.

In the competitions performed in PBMC cultures with PBMC-derived titers, C19 and C27 could replicate and compete with either the r5 (B2 and C3) or the x4 (A8 and E6) viruses (Fig. 3). In Fig. 4, we performed the same competitions as in Fig. 3 but prevented replication of either the “CXCR4”-using component (panel A) or the “CCR5”-using component (panel C) of the dual tropic C19 and C27 viruses. First, the C19+A8, C19+E6, C27+A8, and C27+E6 dual infections were added in equal “x4” titers (determined on U87.CD4.CXCR4 cells) to PBMC cultures in the presence of high maraviroc (MVC) concentrations. As described in Fig. 4b, the IC_99_ concentration of MVC, a CCR5 antagonist [50, 51], resulted in complete inhibition of the r5 B2 and C3 viruses but no apparent inhibition of C19 and C27 in PBMCs. Lack of C19 and C27 inhibition by MVC suggests that the majority of the HIV-1 clones in these quasispecies may be dual tropic (r5/x4) and fully capable of infecting CXCR4+ cells in the PBMC cultures such that MVC does not inhibit any virus replication. The composition of the quaispecies in terms of co-receptor usage is described below (Fig. 1). Interestingly, there was no significant difference in the replicative fitness of C19 or C27 (versus control x4 A8 or E6 HIV-1) when comparing results of competitions performed in PBMCs, PBMCs + MVC, or in U87.CD4.CXCR4 cells (Fig. 4a). The latter condition would only support replication of the control x4 viruses and the “CXCR4”-using component of the dual tropic viruses. The reference viruses A8 and E6 were significantly more fit than the “CXCR4”-using component of the dual tropic C19 and C27 viruses regardless of the above conditions (Fig. 3a).

To limit replication to only the “CCR5”-using component of the dual tropic viruses, we performed competitions in PBMCs + AMD3100 or in U87.CD4.CCR5 cell cultures with the “r5” titers of these viruses. AMD3100, a CXCR4 antagonist [52, 53], showed strong inhibition of x4 A8 and E6 viruses as well as the dual tropic C19 and C27 viruses whereas the r5 B2 and C3 viruses were largely unaffected (Fig. 4d). Reduction in B2 and C3 replication, as compared to no-drug controls, relates to the cellular toxicity from high AMD3100 concentrations, i.e. required for complete inhibition of x4 virus replication but this did not have an effect on the competition experiments as later shown with the B12 and B19 viruses. Whereas the dual tropic C19 and C27 could easily out replicate the r5 B2 and C3 viruses in PBMC cultures, the “CCR5”-using component of these viruses were significantly less fit than B2 and C3 (Fig. 4c). This data is consistent with the relatively weak fitness of r5 subtype C HIV-1 isolates [38–40, 49]. Again these were based only on the r5 phenotype present as r5 or r5/x4 clones in the quasispecies in C19 and C27. Thus, use of AMD3100 with PBMCs or U87.CD4.CCR5 cells would limit replication to only the CCR5-using and r5/x4 components on CCR5+ cells. In PBMCs (without drug) where the “CXCR4” component was not inhibited, the C19 and C27 were 50- to 100-fold more fit than the r5 B2 and C3 controls. After eliminating replication of the pure CXCR4-component with AMD3100, the r5 competitors, B2 and C3 were now significantly more fit than the “CCR5”-using phenotype of the dual tropic C19 and C27 viruses (Fig. 4c). The 10,000-fold shift in fitness when inhibiting the “CXCR4”-using component of the primary C19 and C27 isolates (Fig. 4c) would suggest that the majority of these dual mixed virus populations were comprised of dual tropic clones (e.g. clones that use either CXCR4 or CCR5 for entry, thus r5x4) and that the “CXCR4”-using phenotype (in absence of inhibition) was dominant in determining fitness.

### Co-receptor usage of the C19 and C27 quasispecies

The predicted amino acid sequences of the V3 loop of each C19 and C27 clone (Fig. 2) were aligned and then used in various algorithms to predict co-receptor usage. Based on the C27 consensus sequence, five “groups” of C19 clones were identified based on a unique V3 loop amino acid profile (Additional file 1: Fig. S1). There were three “groups” of clones in the C27 isolate. The five C19 groups all had Ser and Lys at position 11 and 25 and were predicted x4 by the 11/25 rule (positive charge at either or both sites) whereas two neutral amino acids, G11 and A25 predicted a pure r5 phenotype in all of the C27 clones (Additional file 1: Fig. S1). Using the PSSM [54], Geno2Pheno algorithms [25], and PhenoSeq [55], all C19 and C27 clones were predicted to use at least CXCR4 for entry and that CXCR4-using clones may dominate the virus population (PSSM predicted a possible dual tropism) (Additional file 1: Fig. S1).

**Fig. 5:**
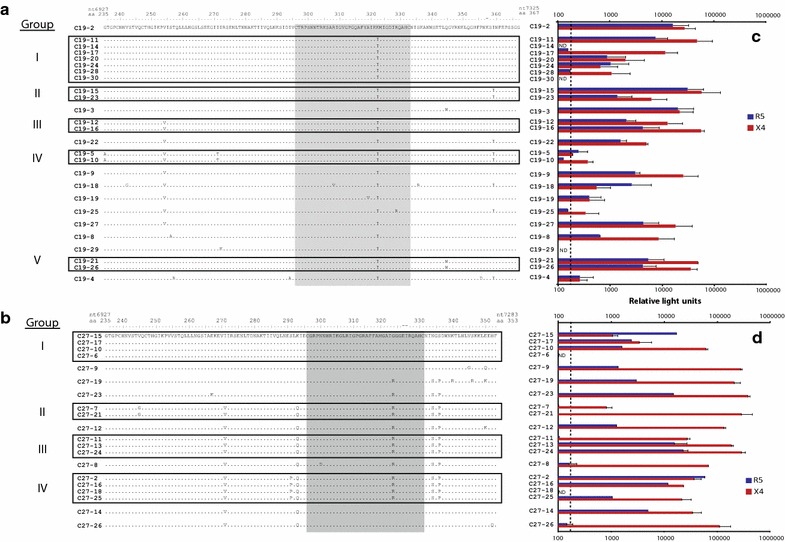
Determining the co-receptor usage of C19 and C27 Env clones introduced into HIV-1 NL4-3 backbone via yeast-based recombination. The 26 C19 and 21 C27 env regions (same as those sequenced for Fig. 1) were cloned into pREC_env/URA3 via yeast-based recombination/gap repair [81, 82]. A 132 and 118 amino acid sequence of the C2-V3 region was used to group the C19 (**a**) and C27 (**b**) based on sequence identity/difference. The pREC_env expression vector was then used to pseudotype virus produced from 293T cells co-transfected with the pNL luc-AM vector [56]. Equal virus titers (based on RT activity) were used to infect U87.CD4 cells expressing either CCR5 or CXCR4. The infecting virus is reverse transcribed and integrated to express luciferase but is incapable of subsequent rounds of infection [56]. Luciferase activity (relative light units) was measured from lysed cells collected 72 h post infection with both the C19 (**c**) and C27 (**d**) Env pseudotyped virus infections. All assays were performed in triplicate. Background was subtracted from results and *dotted lines* on **c**, **d** represent the value three times the standard deviation of the background.

The predicted co-receptor usage was then compared to actual co-receptor usage of the HIV-1 clones from C19 and C27. The HIV-1 Env genes sequenced in Fig. 2a and used for co-receptor prediction in Additional file 1: Fig. S1, were introduced into pREC_nfl_HIV-1_nl4-3_Δenv/URA3 via yeast-based recombination and described above [42]. This Env expression vector was then used to pseudotype virus produced from 293T cells co-transfected with the pNL luc-AM vector [56]. Equal virus titers (based on RT activity) were used to infect U87.CD4 cells expressing either CCR5 or CXCR4. Three (C19-14, C19-30, and C19-29) of 26 C19 clones and 2 of 20 C27 env clones were non-functional. Of the functional clones, none of the C19 or C27 clones were purely CCR5 tropic whereas several clones were “pure” CXCR4-using, i.e. C19-17, -28, -10, -25 (Fig. 5c) and C27-7, -21, -8, -26 (Fig. 5d).

For these analyses, we sequenced approximately 500 nt for each clone and identified 15 unique C19 Env sequence patterns, five of these were described as “groups” I–V (Fig. 5a). Eleven unique sequences and four “groups” (I–IV) were identified in the C27 HIV-1 isolates (Fig. 5b). In general, the clones within the same group (sharing the same C2-V3 nt sequence) showed similar co-receptor usage profiles (compare Fig. 5a with c; b with d). However, this was not always the case. For example in group I, C19-17 and C19-28 appeared to be pure x4 tropic whereas clones C19-11, -20, and -24 could infect both CCR5 and CXCR4 expressing cells (r5x4). It is important to note that clones that share sequence identity in the C2-V3 region may still differ in the remaining ~2,200 nt of Env (not sequenced), specifically in the V1V2 region which has been shown to influence co-receptor usage. Nonetheless, similar co-receptor usage was observed for most clones that share at least the C2-V3 sequences.

Finally, we compared five assays to determine co-receptor usage of these primary isolates: (1) the relative infection by primary C19 and C27 HIV-1 isolate on U87.CD4.CCR5 cells and U87.CD4.CXCR4 cells (Fig. 1c, d), (2) the TCID_50_ values derived on CCR5+ and CXCR4+ cells, (3) the relative inhibition by AMD3100 and MVC on PBMCs (Fig. 4b, d), (4) predicted co-receptor usage from the clones (Additional file 1: Fig. S1), and (5) the actual co-receptor usage of Env clones in the quasispecies (Fig. 5c, d). For these two dual tropic HIV-1 isolates, the vast majority of clones in the quasispecies were used both R5 and X4 co-receptors. As discussed below, the use of co-receptor antagonist coupled with the TCID_50_ measurements on U87.CD4.CCR5 cells and U87.CD4.CXCR4 cells provides the best prediction of the co-receptor usage within the HIV-1 quasispecies. The C19 and C27 primary HIV-1 isolates achieved a dual/mixed phenotype through similar quasispecies compositions. Both had more clones using both co-receptors, very few using only CXCR4, and no pure r5 clones (Fig. 5c, d). As described in our fitness analyses, the CXCR4 usage phenotype of a dual tropic virus is largely dominant in replicative fitness such that when it is inhibited, there is a total loss in fitness of these isolates.

### Contribution of the CXCR4 and CCR5 using variants to replicative fitness in env chimeric viruses

**Fig. 6:**
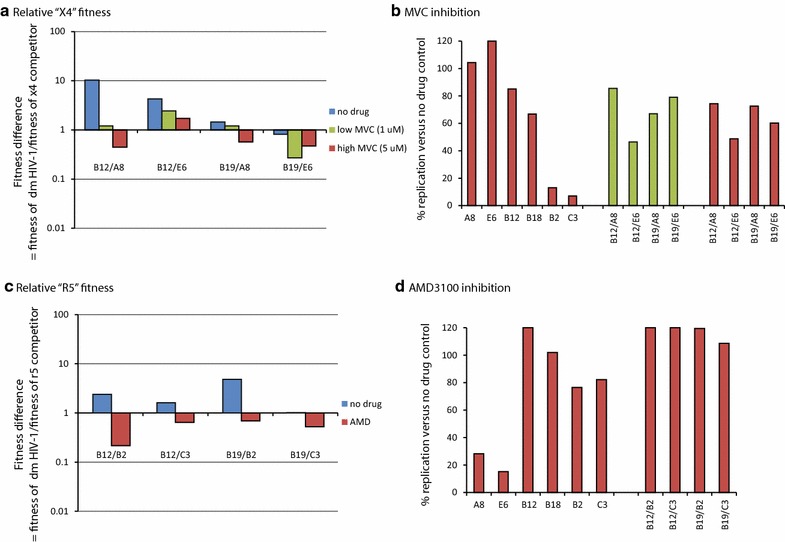
Fitness of the dual tropic env chimeric virus when blocking the virus component using CCR5 or CXCR4. **a** The dm env **c**himeric viruses (B12 and B19) were competed against the x4 reference viruses (all titered on U87.CD4.CXCR4 cells) in U87.CD4.CXCR4 cultures or in PHA-stimulated, IL-2 treated PBMC cultures with or without maraviroc. The fitness differences are plotted as described in Fig. 2 and “Methods”. **b** The level of maraviroc inhibition of the monoinfections or dual infections was measured by relative RT activity in the cell free supernatant and plotted as a percentage of the no drug control. **c** The env chimeric HIV-1 and r5 reference strains (B2 and C3) were titered on U87.CD4.CCR5 cells and then competed together in PHA-stimulated, IL-2 treated PBMC cultures with or without AMD3100. The same dual infection with the same MOI (0.004) was repeated in U87.CD4.CCR5 cultures. The fitness differences are plotted as described in Fig. 2 and “Methods”. **d** The level of AMD3100 inhibition of the monoinfections or dual infections was measured by relative RT activity in the cell free supernatant and plotted as a percentage of the no drug control. All experiments were performed in triplicate with the exception of **c**, **d** (performed in duplicate) due to limited supply Of AMD3100.

Predominance of the x4 and r5x4 clones in the C19 and C27 primary HIV-1 isolates may be related to inability of r5 clones to compete during propagation of these primary HIV-1 isolates (as previously described; [57]). As described above, we also identified two patients infected with dual tropic HIV-1 through env chimeric virus construction [42]. By this method, there is no propagation to result in selection. Bias in the HIV-1 env quasispecies composition could be introduced during PCR and cloning. However, the targets for primer annealing and yeast recombination on the HIV-1 genome were conserved for all HIV-1 strains [47]. In contrast to the C19 and C27 primary isolates, the B12 and B29 env chimeric viruses had higher r5 than x4 titers (Fig. 1e). Thus, there were minimal adjustments for equal “r5” titers and relative fitness of B12 and B19 env chimeric viruses were similar to B2 and C3. AMD3100 had minimal inhibition of B12 and B19 (adjusted for equal r5 titers) (Fig. 6d), which was expected based on the low x4-using component in the viral populations. The inhibition of “CXCR4”-using component (with AMD3100) (Fig. 6c) resulted in only a slight reduction in replicative fitness of the dual tropic B12 and B19 env chimeric viruses. This 2- to 10-fold decreased fitness in B12 and B19 with AMD3100 treatment was minimal compared to 10,000-fold loss in C19 and C27 fitness under the same conditions.

Due to the low “CXCR4”-using component levels in the B19 virus, equalizing x4 titers required the addition of >1,000-fold more volume of B19 (compared to B12) in competition with x4 control strains A8 and E6. Even with this high concentration of B19 virus over the A8 and E6 viruses, the B19 had only slightly greater fitness than A8 and equal fitness to E6 viruses whereas the B12 virus was slightly more fit than both A8 (by tenfold) and E6 (by threefold) in PBMCs (Fig. 6a). Inhibition by MVC appeared to be masked by very high levels of virus needed to reach equalized titers of the “CXCR4”-using component (Fig. 6b). This r5 HIV-1 concentration likely saturated the susceptible CCR5+/CD4+ cells in the PBMC culture. Nonetheless, some inhibition of the more abundant “CCR5”-using component in the B12 and B19 viruses did reduce the overall fitness of B12 and B19 viruses compared to the A8 and E6 viruses (Fig. 6a). By reducing the MVC concentration, the B12 and B19 viruses regained fitness which again suggests that the “CCR5”-using component is contributing to the relative production of B12 and B19 in these competitions.

### Prediction of low level x4 usage

With the B12 and B19 primary HIV-1 isolates, use of the co-receptor antagonists coupled with the TCID_50_ measurements on U87.CD4.CCR5 cells and U87.CD4.CXCR4 cells provided the best prediction of co-receptor usage within the HIV-1 quasispecies. Thus, the x4-using or r5x4 component of the B12 and B19 chimeric env viruses was less than 1 and 0.1 % of the r5-using component, respectively. Based on the estimated low frequency of x4 or r5x4 clones, phenotypic analyses of co-receptor usage would require the cloning and analysis of >100 clones for B12 and >1,000 B19 clones (Table 1a, b) to identify r5 clones. Instead, we compared the predicted co-receptor usage for the B19 HIV-1 population found in: (1) env RT-PCR product from the original patient plasma sample, (2) in the pREC_nfl_envB12 plasmid population following yeast-based cloning of the B19 env PCR product, and (3) in the HIV-1 envB12 chimeric virus populations produced from 293T cells co-transfected with the pREC_nfl_envB12 + pCMV_cplt (see [42] for methods). The viral load in the B12 and B19 plasma samples was >50,000 copies/ml. Over 5,000 copies of RNA (from plasma and env chimeric virus) was reverse transcribed and then PCR amplified for subsequent 454 pyrosequencing of the V3 loop as described in the “Methods”. Over 100,000 copies of DNA plasmid was sampled for 454. Unfortunately, the RT-PCR products of the B19 plasma sample (58,000 copies/ml) were unavailable for these analyses. An average depth of ~1,500–4,000 reads were obtained per sample. Only 105 nt of the V3 loop were analyzed and used to predict co-receptor usage based on 11/25 rule, subtype specific PSSM, Geno2Pheno (G2P, with a 5 % FPR), and PhenoSeq (Table 1a, b). For B19 plasma sample, 95 % of the 2,363 V3 reads were identical and predicted to be r5 by all three algorithms. Approximately 2 % of the reads (N = 51) had Lys at amino acid position 25 in the V3 loop and were predicted by PSSM and G2P to be x4 (Table 1a). Consistent with the low titers of x4 versus r5 in this B19 env chimeric virus (<0.1 %), we could not detect an x4 clone in ~4,100 V3 reads from the plasmid or the resulting virus. For B12 env chimeric virus, 10 clones (0.3 %) were predicted as x4 using by G2P but only 6 of these carried Lys at position 25 and were x4 using based on PSSM (Table 1b). Again, the percentage of predicted x4 or dual tropic clones in the B12 chimeric virus population was consistent with the relative x4 and r5 titers (Fig. 1e).

**Table 1:** Determination of predicted co-receptor usage in the HIV-1 isolate by 454 deep sequencing analyses

Culture	N	Plasmid	N	Plasma	N	Genotype		PSSM results			Geno2Pheno (5 %)	
						11/25	Prediction	% X4	% R5	Prediction	FPR (%)	Prediction
Predicted co-receptor usage for B19 clones^a^												
B19 C1	4,070	B19 P1	4,102	B19 R1	2,237	S/E	CCR5	0.11	0.62	CCR5 only	33.90	CCR5
B19 C2	25					S/E	CCR5	0.11	0.59	CCR5 only	10.90	CCR5
B19 C3	5					S/E	CCR5	0	0.1	CCR5 only	55	CCR5
B19 C4	3					S/E	CCR5	0.22	0.82	CCR5 only	41.20	CCR5
		B19 P2	3			S/E	CCR5	0.22	0.89	CCR5 only	50.50	CCR5
				*B19 R2*	*51*	*S/K*	*CXCR4*	0.24	0.92	CCR5 only	7.80	CCR5
				B19 R3	13	S/E	CCR5	0.22	0.86	CCR5 only	26.90	CCR5
				B19 R4	11	S/E	CCR5	0.11	0.42	CCR5 only	72	CCR5
				B19 R5	7	S/E	CCR5	0.11	0.46	CCR5 only	47.70	CCR5
				B19 R6	7	S/E	CCR5	0.11	0.46	CCR5 only	47.70	CCR5
				B19 R7	6	S/E	CCR5	0.11	0.46	CCR5 only	47.70	CCR5
				B19 R8	6	S/E	CCR5	0.11	0.46	CCR5 only	47.70	CCR5
				B19 R9	6	S/E	CCR5	0.11	0.46	CCR5 only	47.70	CCR5
				B19 R10	5	S/E	CCR5	0.11	0.6	CCR5 only	42.30	CCR5
				B19 R11	4	S/E	CCR5	0.11	0.47	CCR5 only	18.20	CCR5
				B19 R12	4	S/E	CCR5	0.09	0.39	CCR5 only	59.80	CCR5
				B19 R13	3	S/E	CCR5	0.11	0.46	CCR5 only	47.70	CCR5
				*B19 R14*	*3*	*S/K*	*CXCR4*	*0.29*	*0.95*	*CXCR4 capable*	*3.20*	*CXCR4*

Plasmid	N	Culture	N	Genotype		PSSM Results			Geno2Pheno (5 %)	
				11/25	Prediction	% X4	% R5	Prediction	FPR (%)	Prediction
Predicted co-receptor usage for B12 clones^a^										
B12_P1	3,611	B12_C1	1,405	S/Q	CCR5	0.31	0.96	CCR5 only	20.40	CCR5
B12_P2	55	B12_C2	19	S/Q	CCR5	0.31	0.96	CCR5 only	20.40	CCR5
B12_P3	24	B12_C3	10	S/Q	CCR5	0.31	0.96	CCR5 only	20.4	CCR5
B12_P4	21	B12_C4	6	S/Q	CCR5	0.27	0.95	CCR5 only	18.80	CCR5
		B12_C5	6	S/Q	CCR5	0.33	0.96	CCR5 only	22.60	CCR5
		B12_C6	6	S/Q	CCR5	0.31	0.96	CCR5 only	20.40	CCR5
		B12_C7	5	S/Q	CCR5	0.36	0.98	CCR5 only	10.50	CCR5
		B12_C8	5	S/Q	CCR5	0.36	0.98	CCR5 only	17.10	CCR5
B12_P10	3	B12_C9	5	S/Q	CCR5	0.33	0.96	CCR5 only	20.20	CCR5
B12_P17	3	B12_C10	4	S/Q	CCR5	0.31	0.96	CCR5 only	20.40	CCR5
		B12_C11	4	S/Q	CCR5	0.47	0.98	CCR5 only	36.90	CCR5
B12_P15	3	B12_C12	3	S/Q	CCR5	0.31	0.96	CCR5 only	20.40	CCR5
		B12_C13	3	S/Q	CCR5	0.51	0.98	CCR5 only	35.60	CCR5
B12_P8	4	B12_C14	3	S/Q	CCR5	0.33	0.96	CCR5 only	49.70	CCR5
		B12_C5	3	S/Q	CCR5	0.33	0.98	CCR5 only	14.40	CCR5
B12_P5	7	B12_C1	1,405	S/Q	CCR5	0.31	0.96	CCR5 only	20.40	CCR5
*B12_P6*	*6*	B12_C2	19	*S/K*	*CXCR4*	*0.53*	*0.99*	*CXCR4 capable*	*4.80*	*CXCR4*
B12_P7	4	B12_C3	10	S/E	CCR5	0.36	0.98	CCR5 only	*1.70*	*CXCR4*
B12_P9	3	B12_C4	6	S/Q	CCR5	0.29	0.95	CCR5 only	13.10	CCR5
B12_P11	3	B12_C5	6	S/Q	CCR5	0.31	0.96	CCR5 only	20.40	CCR5
B12_P12	3	B12_C6	6	S/Q	CCR5	0.42	0.98	CCR5 only	6.80	CCR5
B12_P13	3	B12_C7	5	S/Q	CCR5	0.31	0.96	CCR5 only	17.80	CCR5
B12_P14	3	B12_C8	5	S/Q	CCR5	0.33	0.96	CCR5 only	7.90	CCR5
B12_P16	3	B12_C9	5	S/Q	CCR5	0.29	0.95	CCR5 only	8.20	CCR5
B12_P18	3	B12_C10	4	S/Q	CCR5	0.51	0.98	CCR5 only	6.80	CCR5

Italics values indicate clones with different amino acids at the 11/25 positions

^a^The C2-V3 coding regions was RT-PCR amplified with barcoded 454 primers from the plasma RNA (only for B12), from the plasmid population following yeast recombination (no RT step), and from RNA of culture supernatant. Amino acid alignments grouped identical clones. Predictive co-receptor algorithms, 11/25, PSSM, and Geno2Pheno were run on the V3 loop amino acid sequences. *FPR* false positive rate for Geno2Pheno at 5 %.

## Discussion

HIV-1 disease progression is associated with the appearance of viral variants that are able to use an expanded repertoire of co-receptors for cell entry. Three phenotypes of HIV-1 isolates have been described: the r5 isolates that are more frequently transmitted and also predominate during the asymptomatic period of infection, the x4 variants that predominate in about 50 % of the infected individuals in the late phase of infection, dm and dual tropic isolates that could represent a transitional phenotype from r5 to x4. The x4, dual tropic and dm viruses have been associated with rapid CD4 declines and progression to AIDS and death but the prevalence/appearance of x4, dm and dual tropic HIV-1 was originally described for subtype B infections [28–31, 36]. A switch to an x4 or dual tropic phenotype in HIV-1 subtype C infections is quite infrequent [58] whereas subtype D HIV-1 infections have much higher prevalence of x4/dual tropic viruses, which also appear earlier in disease [59, 60]. However, Johnston et al. [61] screened 28 subtype C patients and 20 of these were found to be x4 or dual tropic. The high x4/dual tropism in this cohort was attributed to the fact that all these patients had been on antiretroviral therapy and for most of them, there was evidence of resistance. It is also important to note that the propagation of primary HIV-1 isolates on PHA-activated PBMCs commonly enriches for CXCR4-using virus even in HIV-1 populations with a low frequency x4 compared to r5 variants [57]. Early studies on HIV-1 tropism which defined the syncytia inducing (SI) and non-SI phenotypes relied on propagation which may have favored replication of the SI, T cell tropic HIV-1 clones (now defined as x4).

The rate of x4, dm or dual tropic HIV-1 outgrowth during propagations from patient samples typically depends on both the replicative fitness of x4 and r5 HIV-1 clones. This replicative fitness may also be one of many variables contributing to the appearance of x4 viruses during disease progression. As described earlier, other factors may include availability of target cells, co-receptor expression on susceptible cell subsets, differential immune response to x4 and r5 viruses, and preferential transmission of r5 variants followed by a slow evolution to x4 virus. Increased “in vivo” fitness of r5 HIV-1 variants over low frequency x4 variants in the intrapatient HIV-1 population is quite apparent based on various MVC treatment studies [62, 63]. Treatment failure of maraviroc can result from the emergence of resistant CCR5 (r5)-using viruses with reduced susceptibility to MVC [42–45]. However, the most common escape pathway for MVC resistance relates to the outgrowth of the intrinsically resistant x4 variants in a treated individual. In almost all cases, the x4 virus was found within the quasispecies prior to the treatment and often at a frequency requiring sensitive phenotypic or genotypic assays for detection [62, 63]. In the environment of MVC treatment, x4 emerged due to increased fitness over r5 and possibly dual tropic viruses in the intrapatient HIV-1 population. Based on these findings, the relative contribution of r5 and x4 virus components to the overall fitness of a primary isolate as well as the emergence of dual tropic HIV-1 isolate may have important implications on subsequent disease progression or response to MVC treatment.

In this study, we have measured the ex vivo replicative fitness of two dual tropic subtype C HIV-1 isolates and two dual tropic env chimeric viruses under different conditions. In our previous studies on replicative fitness involving pairwise competitions in PBMCs, it was important to standardize the initial virus inoculums via infectious titers, commonly calculated as TCID_50_ values determined with PBMCs from the same donor and blood draw as those used in the competitions [57, 64]. Subsequent dual infections with two equal inocula of viruses of the same phenotype (i.e. r5 vs. r5 viruses or x4 vs. x4 viruses) will then compete for the same susceptible cell population within PBMCs. When dual infections are performed with an r5 and x4 virus, the viruses do not necessarily compete since they replicate in different cell populations within PBMCs and yet, the x4 viruses almost always replicate with faster kinetics to dominate the cultures simply because they have a stronger pathogenic phenotype [41, 57]. Thus, we wanted to assess the relative contribution of the CXCR4 and CCR5 components to the replicative fitness of dual tropic HIV-1 strains. We first screened for dual tropism in a collection of primary HIV-1 isolates and in env chimeric HIV-1 isolates constructed from patient plasma samples. We obtained two dual tropic primary HIV-1 isolates (C19 and C27) and two dual tropic env chimeric virus constructs (B12 and B19).

Co-cultivation of HIV + PBMCs with PBMCs derived from HIV-negative donors is a common method to obtain primary HIV-1 isolates. Several studies have indicated that x4 or dual tropic clones can expand more rapidly than r5 clones [57, 65]. As a consequence, x4 clones may predominate the primary HIV-1 isolates even if the original HIV-1 quasispecies in the patient PBMCs had very low frequency of x4 versus r5 clones. When we have examined the x4 frequency in several primary dual tropic HIV-1 isolates, the x4 or r5x4 clones typically predominate the quasispecies (as with C19 and C27) [57]. Because of this selection bias during virus propagation, we now clone the env gene from patient samples into a plasmid with the HIV-1 NL4-3 backbone and then produce virus by direct DNA transfection. Typical bacterial cloning of ~3 kbp fragment into a ~8 kbp DNA vector is quite inefficient, produces few bacterial colonies containing the plasmid with insert, and results in an undesired, severe genetic bottleneck on the HIV-1 env quasispecies. As previously described, cloning of this env product into the pREC_nfl_HIV-1_NL4-3_Δgp120/URA3 construct via yeast homologous recombination/gap repair results in thousands of yeast colonies and a minimal genetic bottleneck in the env quasispecies [47]. Nonetheless, complementation with the patient-derived gp120 coding region (or entire env gene) and the NL4-3 backbone can occasionally result in a “dead” virus [66].

Using the primary HIV-1 isolates and the env chimeric viruses for fitness studies, we observed significant differences that reflected the r5, x4, and r5x4 composition in the quasispecies. Both x4r5 and x4 clones were observed in C19 and C27 primary HIV-1 isolates (1) as predicted by co-receptor usage algorithms applied to clonal sequencing analyses and (2) as determined by a phenotypic co-receptor usage assay applied to HIV-1 clones in the quasispecies. Interestingly, the “CXCR4”-using component was dominant over the “CCR5”-using component in determining replicative fitness of the r5x4 population in the C19 and C27 primary isolates (pure x4 clones were rare and r5 clones were absent). When the “CXCR4”-using component was completely inhibited in these primary HIV-1 isolates, the “CCR5”-using component of the r5x4 population could not compete with control r5 HIV-1 isolates. Interestingly, when competitions were limited to the “CCR5”- or “CXCR4”-using component in the primary r5x4 HIV-1 isolate, the replicative fitness was quite similar to related primary r5 and x4 HIV-1 isolates. The r5- and x4-using components of the C19 and C27 viruses were significantly less fit than the r5 B2 and C3 HIV-1 isolates or the x4 A8 and E6 HIV-1 isolates, respectively. We have published several studies showing that primary subtype C HIV-1 isolates are less fit than any other group M HIV-1 isolate (determined in head-to-head competitions in PBMCs from multiple donors) [38–40, 49]. It is interesting to note that from our unpublished and published data, subtype C competes well with other subtypes in ex vivo competition in langerhans cells implying that the transmission fitness of this subtype is comparable to that of other subtypes. However, after entry into cells, the pathogenic fitness of subtype C is lower than other subtypes, a possible explanation of the dominance of r5 tropism in subtype C which leads to a slower disease progression. In general, the less virulent r5 variants are more common during late subtype C infection.

Based on previous studies [67, 68] it appears that dual tropic isolates may be evolutionary intermediates. The early phase of intrapatient HIV-1 evolution consists predominantly of r5 variants possibly with a few r5x4 but likely minimal or no x4 variants [57, 69]. During the time of infection and possibly the absence of treatment, the r5x4 variants may increase in frequency and possibly mutate to a pure x4 variant [65, 70, 71]. However, previous studies also suggest that these r5x4 and x4 variants often represent separate lineages from CCR5 variants with deep roots within the intrapatient phylogenetic tree [72]. The actual mutational pathways from a single HIV-1 clone that leads to a phenotypic switch from r5 to x4 may arise with significant cost to replicative fitness [73]. These findings imply that this shift in tropism may be under selective pressure, i.e. increased availability of CXCR4+ versus CCR5+ susceptible cells. In our studies it appears that the “CXCR4”-using component of the r5x4 clones in the C19 and C27 were dominant over the “CCR5”-using component in determining replicative fitness. The “CXCR4”-using component of the dual tropic C19 and C27 HIV-1 isolates were generally less fit than the control x4 A8 and E6 HIV-1 isolates. However, we cannot assume that this reduced fitness was due to the initial mutation pattern leading to a switch in r5 to x4 since subtype C HIV are generally less fit than any other group M HIV-1 isolate.

The findings with C19 and C27 primary HIV-1 isolates might suggest that the “CXCR4”-using component of a dual tropic HIV-1 would dominate over the “CCR5”-using component in replicative fitness. However, this finding is misleading for patients harboring mixed X4 and R5 HIV viruses. As noted earlier, the primary HIV-1 isolates such as C19 and C27 primarily contain x4 or r5x4 clones and only low levels of r5 clones due to selection during PBMC co-cultivation/propagation. Within patients, x4 or r5x4 HIV-1 are often found at low frequency in the quasispecies. Thus, we constructed the B12 and B19 env chimeric viruses from patients with mixed X4 and R5 HIV-1. In these viruses, the r5 clones were 100- and 1,000-fold more abundant than the x4 or r5x4 clones. These findings were confirmed by (1) measuring infectious titers in both U87.CD4.CCR5 cells and U87.CD4.CXCR4 cells and by (2) predicting co-receptor usage from approximately 1,000–4,000 V3 loop sequence reads (derived by 454 pyrosequencing of ~10,000 patient-derived DNA templates). In the B12 and B19 env chimeric viruses, the CCR5 component derived from the pure r5 clones did contribute to overall replicative fitness. The more dominant “CXCR4” component in the C19 and C27 resulted in the ability to compete with the control x4 A8 and E6 HIV-1. In contrast, high levels of “CCR5” versus the “CXCR4” component in the B12 and B19 env chimeric virus resulted in low replicative fitness when competed against x4 A8 and E6 HIV-1. In general, x4 HIV-1 out replicates r5 HIV-1 in PBMC cultures. When adjusting for the r5 or x4 titers, the “r5” or “x4” components of B12 and B19 HIV-1 had similar fitness and were able to compete with the r5 (B2 and C3) and x4 (A8 and E6) control strains. These findings are consistent with our previous studies in that subtype B x4 or r5 HIV-1 isolates have, on average, higher replicative fitness than HIV-1 isolates from other group M subtypes [38–40].

Previous studies suggest a dominance of the “CXCR4”-using over the “CCR5”-using phenotype in HIV-1 defined as dual tropic/mixed tropic. As a consequence, a proposed x4 dominance in mixed X4 and R5 HIV-1 has been implicated in MVC treatment outcome and disease progression. Again, our findings with the C19 and C27 viruses suggest that use of primary HIV-1 isolates may over-emphasize this x4 dominance. The x4 or x4r5 HIV clones are typically dominant within the intrapatient quasispecies of some patients during late infection [65, 74]. Otherwise, x4 or x4r5 HIV clones are found at low frequencies compared to the pure r5 clones in the intrapatient quasispecies during asymptomatic disease. Furthermore, “CXCR4”- using HIV isolates are rare in subtype A and C [27, 58, 75] but more frequent in subtype B and D infected patients [59]. The x4 HIV strains typically have faster replication kinetics than r5 HIV but due to limited CCR5+/CXCR4+/CD4+ cells in PBMC cultures, these two HIV-1 phenotypes are not in direct competition in our studies. When we equalize for r5 or x4 titers in the dual tropic HIV-1 and limited replication to the “CCR5”-using and “CXCR4”-using components, we found that fitness of C19 and C27 virus reflected that of other subtype C HIV-1 isolates, i.e. less fit than most group M HIV-1 isolates. In contrast, even the low level of x4 or x4/r5 B12 and B19 HIV-1 had replicative fitness comparable to the control x4 viruses. However, without adjusting virus titers for the “CXCR4-using” component, this low level of x4 HIV-1 had minimal impact on replicative fitness of this env chimeric virus which was dominated by r5 clones. These findings suggest that detection of low frequency x4 clones in the HIV-1 quasipecies is not necessarily synonymous with rapid HIV replication kinetics and faster disease progressions. However, the overall replicative fitness in PBMCs regardless of the x4 and r5 composition remains a strong correlation of disease progression [41, 57, 76–78].

## Conclusions

In this study, we show that x4 component is dominant in replicative fitness but is typically of low frequency compared to r5 HIV-1 in most dual tropic HIV-1 quasispecies. Based on findings in this study, the emergence of dual/mixed tropic viruses may have important implications on response to maraviroc containing regimens. Furthermore, although the “CXCR4”-using phenotype is of low frequency in the dual/mixed population, it may have a selective advantage in patients failing maraviroc. Based on our findings that the “CXCR4”-using phenotype is the major determinant of replicative fitness, this may have important implications on disease progression.

## Methods

### Cells

293T cells (human embryonic kidney cells) were grown on Dulbeco’s Modified Eagle’s medium (DMEM) supplemented with 10 % fetal bovine serum (FBS) (Cellgro), 100 U/ml of penicillin and 100 μg/ml of streptomycin (Cellgro). U87.CD4 (human glioma cells) expressing either CCR5 or CXCR4, obtained from the NIH AIDS Research and Reference Reagent Program (distributed by Dr. D Littman) were maintained in complete DMEM medium supplemented with 1 mg/ml of geneticin G418 (Life Technologies, Inc.) and 5 µg/ml puromycin to maintain receptor and coreceptor expression [5]. Peripheral blood mononuclear cells (PBMCs) from HIV-negative blood donors were obtained by Ficoll-Hypague density centrifugation of heparin treated venous blood. These were stimulated with 2 µg/ml of phytohemagglutinin (PHA; Gibco BRL) and 1 µg/ml of interleukin-2 (IL-2; Gibco BRL) in complete RPMI 1640 containing 2 mM l-glutamine for 3–4 days.

### Virus propagation, chimeric virus production, and titers

Subtype C syncytium inducing (SI) HIV-1 isolates obtained from patients who had been on reverse transcriptase inhibitors for an average of 14.4 months [46] as well as the non-SI and SI-using reference viruses [38, 40] (Table 1) were grown in PHA-activated PBMCs over a period of 2 weeks. RT assays were performed at different time points to measure virus production. As determined by Abraha et al. [40], co-receptor usage was then estimated by exposing U87.CD4.CCR5 and CXCR4 cells to each virus.

In a subtype B cohort analyses, RNA was extracted from plasma samples from 11 untreated, HIV-infected patients (investigators were blinded to all patient demographics). The HIV-1 env gene was then reverse transcribed-PCR amplified from the plasma RNA with the Env-End primer as described [44]. The gp120 regions were then PCR amplified using primers F_gp120 (5′-GACAGGTTAATTGATAGACTA-3′) and B_gp120 (5′-CTTCCTGCTGCTCCCAAGAAC-3′). Briefly, these gp120 PCR products were cotransformed with SacII-linearized pREC_NFL_Δgp120/URA3 into Saccharomyces cerevisiae MYA-906 cells (ATCC). Following homologous recombination, plasmids were extracted from the yeast cells and transformed into electro-competent Escherichia coli Stbl4 cells (Invitrogen). Individual bacterial colonies were grown, and plasmids were extracted using Qiagen miniprep kits. The cloning procedures have been extensively described [44, 47]. Of the 11 plasma samples from HIV-infected patients, we obtained 3 pREC_nfl_gp120 vectors harboring a functional HIV-1 env gene from the patient. The pREC_nfl_gp120 vectors were then co-transfected into 293T cells along with the complementing vector, pCMV_cplt to produce infectious virus as described [47]. To avoid confusion, we will refer to the viruses with an NL4-3 backbone and containing the patient’s gp120 coding region as env chimeric viruses.

TCID_50_ determination for all the viruses was performed on PHA/IL-2 stimulated HIV negative donor PBMCs as well as on U87.CD4.CCR5 and CXCR4 cells. For PBMCs, 100,000 cells were added to each of the wells in a 96 well plate. Ten-fold serial dilutions of each virus were made and 100 μl were added to each well containing the cells with each dilution performed in triplicates [64]. The viruses were grown in RPMI-1640 complete media. Similar dilutions were made for the U87 cells, 10,000 cells per well were plated in 96 well plates in DMEM complete media containing G418 and puromycin 24 h prior to infection. At the time of infection, media containing selection antibiotics was removed and replaced with DMEM with no selection. Supernatants were harvested at different time points to measure RT activity. TCID_50_ for each virus was calculated using the Reed and Muech method [79].

### Veritrop cell-to-cell fusion assay

A new cell fusion assay to measure co-receptor usage to a 2 % level has been recently described [48]. Briefly, 2 ug of pREC_nfl_env_ptX_ is transfected into 293T cells using Fugene as described [48]. After 24 h, the transfection reagent is removed by cell washing and the 293T cells are layered over U87.CD4.CCR5 and CXCR4 cells transfected with pDM1.1 [80]. Luciferase is only produced from pDM1.1 in the presence of Tat and Rev derived from the 293T cells fused with U87 cells transfected with the pREC_nfl vector, i.e. expressing the entire HIV-1 proteome.

### Determination of fitness of the dual tropic isolates

Fitness of the dual tropic isolates was determined on both PBMCs and U87.CD4.CCR5 and CXCR4 cells using dual competition assays described previously [38–41, 57]. Briefly, for PBMCs, to determine the effect of the “CCR5”-using phenotype on fitness, the dual-tropic isolates were competed against r5-using isolates (C3 and B2) at a multiplicity of infection (MOI) of 0.004 in the presence or absence of AMD3100, a CXCR4 coreceptor antagonist. AMD3100 was used at a concentration of 0.1 µM (>the inhibitory dose for 99 % inhibition or IC_99_). To determine the role of the “CXCR4”-using phenotype in fitness of the dual tropic isolates, the dual-tropic isolates were competed against x4-using isolates (E6 and A8) in the presence or absence of maraviroc at an MOI of 0.004. Maraviroc was used at a concentration of 1 µM (IC_99_).

On U87 cells, the role of the “CCR5” phenotype on fitness of the dual tropic isolates was determined by competing the dual tropic isolates against the r5 using competitor (B2 and C3) viruses on U87.CD4.CCR5 cells at an MOI of 0.004. The same isolates were competed against the x4-using competitor viruses (E6 and A8) on U87.CD4.CXCR4 cells at an MOI of 0.004. For all the competition experiments, mono-infections representing each of the viruses in the competition were included. Virus production was monitored over a period of time by measuring RT activity at different time points. At the time of peak virus production, the cells were harvested and DNA was extracted using the Qiagen DNA extraction kit.

### Determination of viral fitness using heteroduplex tracking assay

To determine viral fitness, the env gene was PCR amplified in the C2-V3 region using a nested PCR. For the first round ENV B (5′-AGAAAGAGCAGAAGACAGTGGCAATGA) and antisense primer ED 14 (5′-TCTTGCCTGGAGCTGTTTGATGCCCCAGAC) were used to amplify a 1,400 bp of the *env* gene. The second round PCR was performed using a sense primer E80 (5′-CCAATTCCCATACATTATTGTG) and an anti-sense primer E125 (5′-CAATTTCTGGGTCCCCTCCTGAGG) amplifying a 480 bp fragment of the C2-V3 region. PCR conditions were as described. The PCR products were run on a 1 % agarose gel stained with 1 % ethidium bromide and viewed under UV illumination. C2-V3 fragment amplified from subtypes D and E with a ^32^P labeled E80 and unlabeled E125 primer were used as probes. To determine the relative virus production for each virus in the competition, a heteroduplex tracking assay (HTA) was used as previously described [38–41]. Heretoduplexes were separated on a 6 % non-denaturing gel and quantified using a Molecular Imager FX (BioRad) phosphoimager. Virus production for each of the virus in the competition (*f*_*o*_) was divided by the initial proportion in the inoculum (*i*_*o*_) to get the relative fitness value (*w* = *f*_*o*_*/i*_*o*_) [38–41]. The relative fitness value of each isolate in the competition is a measure of the fitness difference *W*_*D*_ between the two HIV-1 variants (*W*_*D*_ *=* *W*_*M*_*/W*_*L*_)_,_ where *W*_*M*_ and *W*_*L*_ correspond to the relative fitness of the more and less fit virus.

### Plasmid generation and env sequencing

The env gene (gp160) was PCR amplified from each of the dual-tropic isolates by nested PCR to obtain a 2,750 bp fragment. The first round PCR was amplified using Env A 5′-GGCTTAGGCATCTCCTATGGCAGGAAGAA (5,954–5,982 positions of the HXB2 genome) and Env M 5′-TAGCCCTTCCAGTCCCCCCTTTTCTTTTA (9,068–9,096). The second round PCR was carried out with Kpn-1Env TGTGGGTCACAGTCTATTATGG (6,325–6,346) and Env-End 5′-CTTTTTGACCACTTGCCACCCAT (8,797–8,819) primers. This PCR product was then cloned into a yeast vector by homologous recombination as described elsewhere. Briefly, the PCR amplified gp160 was cloned into a SacII linearized pREC_nfl_HIV-1_nl4-3_env/URA3 vector by homologous recombination as already described [81, 82]. Thirty colonies were picked for each of the dual-tropic isolates. For each of these, the C2-V3 region was sequenced as described above and then analyzed to determine coreceptor usage via the 11/25 rule or with the webPSSM [54] and Geno2Pheno algorithms [25]. Sequences were aligned using ClustalX [83] and MUSCLE [84, 85] followed by phylogenetic tree constructions using TreeView [86].

Pyrosequencing of the B12 and B19 env chimeric viruses was performed on PCR amplicons of the C2-V3 region. Nested PCR products for sequencing were generated using the external products described above as templates with custom designed fusion oligos that contained the 454 adaptor sequences (Roche Lib-A Primer A and Primer B), followed by a 10 basepair Multiplex Identifier (MID) sequence to permit sample pooling, and lastly the HIV-1 template specific oligos E110 and E125 (5′-CTGTTAAATGGCAGTCTAGCAGAA-3′ and 5′-CAATTTCTGGGTCCCCTCCTGAGG-3′, respectively). Samples were quantified by fluorometry with the Quant-iT PicoGreen dsDNA Assay Kit (Life Technologies), pooled in equimolar concentrations, and sequenced on a 454 GS Junior System (Roche Diagnostics) using the GS Junior Titanium Sequencing chemistry. The resulting reads were trimmed to exclude the MIDs and primer sequence, and low-quality reads were filtered using the GS Run Processor according to length and quality scores. Multisequence alignments of reads were constructed using MUSCLE (PMID: 15034147), and all phylogenetic analysis was performed with MEGA5 (PMID: 21546353).

### Pseudotyping single cycle assays

The pNL luc-AM vector described by Pugach et al. [56] was cotransfected along with one of each pREC env clonal plasmid (thirty for each dual tropic isolate) into 293T cells using effectene transfection kit (Qiagen, Valencia, CA, USA). Pseudovirus-containing supernatants were harvested from each well at 72 h post transfection and then used to infect both U87.CD4.CCR5 and U87.CD4.CXCR4 cells in triplicate. After 72 h at 37°C in 5 % CO_2_, the supernatant was removed and cells were washed with 200 μl of phosphate buffered saline (PBS, Cellgro). 100 µl of cellgro lysis buffer was added to lyse the cells. 50 µl of the Luciferin Steady Glo substrate was added to 50 µl of lysate supernatant to measure luciferase activity.

## Additional file

10.1186/s12981-015-0066-7 Characteristics of primary HIV-1 isolates.

## Abbreviations

r5: CCR5-using
x4: CXCR4-using
dm: dual mixed
HIV: human immunodeficiency virus
Env: envelope glycoprotein
PBMC: peripheral blood mononuclear cells
MVC: maraviroc
TCID_50_: infectious dose of 50 % infectivity in tissue culture
