# Supplementary material for: Defining the fitness of HIV-1 isolates with dual/mixed co-receptor usage
Source: AIDS Res Ther. 2015 Oct 3;12:34. doi: 10.1186/s12981-015-0066-7 (PMC4592561; doi:10.1186/s12981-015-0066-7)
Supplement: Supplementary file 1 — 10.1186/s12981-015-0066-7 Characteristics of primary HIV-1 isolates. [file 12981_2015_66_MOESM1_ESM.pdf]

Supplementary Table 1. Characteristics of primary HIV-1 isolates

Nankya et al.

Lab Reference	Country of Origin	Virus ID	gag/env subtype	Co-receptor usage	$TCID_{50}$ (log infectious units/ml)		
					U87.CD4.CCR5	U87.CD4.CXCR4	PBMCs
C3	South Africa	97ZA012	C/C	CCR5	6.0	<1	4.5
C19	Zimbabwe	TCDD04	C/C	CCR5/CXCR4	5.75	6.25	5.0
C27	Zimbabwe	TCDD30	C/C	CCR5/CXCR4	4.0	4.5	3.25
A8	Uganda	92UG029	A/A	CXCR4	<1	5.0	4.5
B2	Brazil	92BR017	B/B	CXCR4	6.0	<1	4.5
E6	Cameroon	CMU06	A/E	CXCR4	<1	4.5	4.0
B12	USA	---	B/B	CCR5/CXCR4	6.25	5.25	5.5
B19	USA	---	B/B	CCR5/CXCR4	5.25	2.25	5.0

**A.** Env V3 loop sequence	**B.** 11/25	**C.** PSSM		**D.** G2P	**E.** PhSeq
amino acid sequence (HXB2 Env numbering)<br>10 20 30<br>....|....| ....--.....| ....|....| ....| 11 25	prediction based on H,R,K at 11/25	CXCR4-using percentage	CCR5-using percentage	Predicted co-receptor usage	Predicted co-receptor usage
C19 clone 2<br>CTRPSNNTRK ***S***ARIGVGPGQAF YAIK***K***MIGDI RQAHC	SK<br>X4/SI	58%	99%	CXCR4	CXCR4
C19 clones 3-5,8-12,14-17,20-24,26-30<br>CTRPSNNTRK ***S***ARIGVGPGQAF YAIK***K***IIGDI RQAHC	SK<br>X4/SI	51%	98%	CXCR4	CXCR4
C19 clone 18<br>CTRPSNNTRK ***S***ARVGVGPGQAF YAIK***K***IIGDI RQAHC	SK<br>X4/SI	53%	99%	CXCR4	CXCR4
C19 clone 19<br>CTRPSNNTRK ***S***ARIGVGPGQAF YAVK***K***IIGDI RQAHC	SK<br>X4/SI	53%	99%	CXCR4	CXCR4
C19 clone 25<br>CTRPSNNTRK ***S***ARIGVGPGQAF YAIK***K***IIGDI RRAHC	SK<br>X4/SI	58%	99%	CXCR4	CXCR4
10 20 30<br>....|....| ....|....| ....|....-| ....|					
C27 clone 2,7,11-13,15,16,18,19,21,23,24,25<br>CGRPNNHRIK ***G***LRIGPGRAF FAMG***A***IGGGEI RQAHC	GA<br>R5/NSI	80%	100%	CXCR4	CXCR4
C27 clone 6,9,10,14,15,17,26<br>CGRPNNHRIK ***G***LRIGPGRAF FAMG***A***IRGGEI RQAHC	GA<br>R5/NSI	80%	100%	CXCR4	CXCR4
C27 clone 8<br>CGRPDNHRIK ***G***LRIGPGRAF FAMG***A***IRGGEI RQAHC	GA<br>R5/NSI	80%	100%	CXCR4	CXCR4

Supplementary Figure 1. Prediction of co-receptor usage. The C19 and C27 HIV-1 env genes were cloned into pREC_env and then sequenced. The predicted amino acid sequence of the V3 loop (A) was then used to predict co-receptor usage using the 11/25 rule (B), the PSSM (C), Geno2Pheno (D) and the PhenoSeq (E) algorithms.
